# An Interpretable Machine Learning Framework Integrating Multi-Window Environmental Exposure for Hypertension Risk Assessment

**DOI:** 10.3390/toxics14100871

**Published:** 2026-09-30

**Authors:** Ying Zhao, Kexin Yuan

**Affiliations:** 1 Department of Chemistry and Chemical Engineering, Jinzhong University, Jinzhong 030619, China; 2 School of Physical Education, Shanxi University, Taiyuan 030006, China

**Keywords:** machine learning framework, hypertension risk prediction, PM_2.5_, multi-window exposure, model interpretability, environmental health

## Abstract

Machine learning (ML) models for hypertension risk prediction have predominantly relied on traditional demographic and clinical risk factors, often overlooking the temporal heterogeneity of environmental exposures. This study developed and validated an interpretable ML framework that systematically integrates multi-window cumulative PM_2.5_ exposure features for hypertension risk assessment. We designed a modular analytical pipeline comprising five ML algorithms—Generalized Linear Model (GLM), Lasso regression, Decision Tree, Random Forest (RF), and XGBoost—coupled with a three-layer interpretability module (variable importance, SHAP values, and partial dependence plots). The framework ingests traditional risk factors alongside cumulative PM_2.5_ exposure across five temporal windows (0-day, 7-day, 15-day, 30-day, and 60-day). As a validation case, the framework was applied to 2523 participant-visits from the Beijing subsample of the China Health and Nutrition Survey. Analyses were performed at the participant level, systolic and diastolic blood pressure were excluded from the predictors of the hypertension classifiers, and models were validated with person-level and year-based splits. After these corrections the five models showed realistic discrimination, with test AUCs of 0.69–0.77 and Brier scores of 0.17–0.23. Age, body mass index (BMI) and waist circumference were consistently among the most important predictors, and the 60-day PM_2.5_ window was the most important exposure feature. Adding the five PM_2.5_ window features improved test AUC by ≈0.02–0.03 in the ensemble models (*p* = 0.03–0.05). Window-specific adjusted analyses showed inverse associations of PM_2.5_ with hypertension that were stronger for longer windows. The proposed framework provides a reusable interpretable approach for incorporating multi-window environmental exposure data into cardiovascular risk prediction. Its modular design enables adaptation to other environmental exposures, health outcomes, and population cohorts, supporting both risk screening and personalized intervention strategies.

## 1. Introduction

Hypertension is a leading contributor to cardiovascular diseases and mortality worldwide, affecting over 1 billion people and causing 9.4 million deaths annually [1,2]. Its complex, multifactorial etiology involves genetics, lifestyle, dietary habits, and environmental factors [3,4]. In parallel, machine learning (ML) approaches have been increasingly adopted for hypertension risk prediction, offering advantages over traditional statistical methods in handling high-dimensional, heterogeneous data and capturing nonlinear relationships [5,6,7]. However, most existing ML-based hypertension prediction models rely heavily on demographic and clinical features (age, BMI, anthropometric measures), while environmental exposures—particularly air pollution—are rarely incorporated as structured predictor variables. This omission represents a critical gap, given the established role of environmental factors in cardiovascular pathophysiology.

Ambient fine particulate matter (PM_2.5_) is recognized by the World Health Organization as the greatest environmental risk to health [8]. PM_2.5_ exposures can damage vascular endothelial cells, promote atherosclerotic plaque formation, and alter coagulation factor distribution, thereby increasing cardiovascular risk [9]. Critically, the health effects of PM_2.5_ are temporally heterogeneous: short-term exposure (hours to days) has been associated with acute blood pressure elevation [10,11,12,13], while chronic exposure (months to years) may produce distinct and potentially opposing physiological responses through adaptive or cumulative mechanisms [14,15,16]. Despite this evidence, existing ML prediction models typically treat environmental exposure as a single-point or annual-average variable, failing to capture the rich temporal structure that may contain independent predictive information.

Epidemiological studies have documented exposure-window-dependent associations between PM_2.5_ and blood pressure. A meta-analysis by Zhao et al. reported that each 10 ug/m^3^ increment in PM_2.5_ was significantly associated with hypertension incidence (RR = 1.21, 95% CI: 1.07–1.35) [11]. Short-term studies have observed 2–3 mmHg increases in systolic and diastolic blood pressure per 10 ug/m^3^ PM_2.5_ increment within 3–7 days of exposure [12,13]. Conversely, long-term exposure studies have yielded more complex findings: Song et al. reported modest blood pressure elevations (0.19 mmHg SBP per 10 ug/m^3^), while other investigations have noted attenuation or reversal of effects over longer windows, potentially reflecting physiological adaptation or unresolved confounding [15,16]. This temporal heterogeneity underscores the need for analytical approaches that can simultaneously model multiple exposure windows and their interactions with traditional risk factors.

Interpretable machine learning offers a powerful solution to these challenges. Modern ML algorithms—including tree-based ensembles such as Random Forest and XGBoost—can model complex, nonlinear relationships without a priori specification of functional forms. Moreover, model-agnostic interpretability tools, including permutation feature importance, Shapley additive explanations (SHAP), and partial dependence plots (PDPs), enable post-hoc dissection of how each predictor contributes to model outputs, including the direction, magnitude, and interaction patterns of effects [5,6,7]. These techniques are particularly well-suited for disentangling the contributions of highly correlated multi-window exposure variables that would pose collinearity problems in traditional regression frameworks.

Beyond environmental exposures, daily behavioral factors—including physical activity, sleep duration, sedentary behavior, and work-related activity—exert important modifying effects on blood pressure and may interact synergistically with air pollution exposure [17,18,19]. A comprehensive prediction framework should therefore accommodate not only multiple exposure windows but also behavioral covariates and their potential interactions with environmental variables.

The individual ML algorithms used here (GLM/Lasso/RF/XGBoost) and the interpretability tools (SHAP/PDP) are standard methods, and their combination has appeared in earlier environmental analyses [5,6,7]. The contribution of the present work is therefore a framework–level design with three elements, which are not jointly provided by earlier applications: (1) a reusable exposure–window–aware feature–engineering layer that decomposes environmental exposure into five nested cumulative windows (0–, 7–, 15–, 30–, 60– day) by a general date–matching rule; (2) a formalized three–layer interpretability protocol (variable importance, SHAP, PDP) with explicit sensitivity rules for correlated exposure features (single-window models, ablation, consensus ranking) and pre–specified prediction targets (binary hypertension); and (3) operational outputs for environmental–health users, including window–specific exposure–response quantities (odds ratios per 10 µg/m^3^), and formal effect–modification tests, in addition to model discrimination and calibration metrics. The individual algorithms and interpretability tools used here are standard, and their combination has been reported in previous environmental analyses. Table 1 compares this framework with representative previous ones across three dimensions: handling of exposure time–windows, detection of interaction effects, and operationalizability of the outputs. As an initial validation, we applied this framework to the 2011–2015 waves of the China Health and Nutrition Survey (CHNS), a large–scale population–based cohort. The three core objectives were: (1) to evaluate the predictive contribution of multi–window PM_2.5_ features relative to traditional risk factors; (2) to characterize nonlinear and synergistic exposure-response relationships through the interpretability module; and (3) to demonstrate the capacity of the framework to generate actionable insights for personalized risk stratification.

## 2. Methods

### 2.1. Study Population

The CHNS is a large-scale, household-based prospective epidemiological study investigating a range of economic, social, demographic, and health issues. This study included the subsample of CHNS participants residing in Beijing city (urban and peri–urban/rural communities) who were examined in the 2011 or 2015 CHNS wave and for whom complete data on blood pressure, covariates, and PM_2.5_ exposure were available (2154 unique participants contributing 2523 participant–visits; 873 visits from the 2011 wave and 1650 from the 2015 wave). Participants examined in both waves contribute two participant-visits, which are analyzed as repeated cross-sections; all machine-learning splits were performed at the participant level so that no participant appeared in both the training and the test set. Daily PM_2.5_ concentrations were assigned from the Beijing monitoring network to each participant’s examination date. Annual average concentrations of the air pollutant PM_2.5_ in various urban districts of Beijing from 2010 to 2015 were collected from the China Environmental Monitoring Station National Urban Air Quality Real-time Release Platform. These datasets were then integrated to establish a spatial database for framework validation.

Related data were collected using a baseline survey questionnaire, comprising household, individual, and activity–specific sections. The household questionnaire primarily covered residents’ living environments and household equipment. The individual questionnaire gathered demographic data, family background, smoking and drinking habits, physical injuries, and psychological status. The activity questionnaire recorded activities related to work, transportation, recreation, and sports. By thoroughly inquiring about participants’ specific activities in the previous week, including their total daily duration, number of days, and whether they were vigorous, moderate, or light, activity values could be calculated.

### 2.2. Definition of Hypertension and PM_2.5_ Exposure

Prior to blood pressure assessment, participants sat quietly for 10 min. Afterward, arterial blood pressure in the right arm was measured three times using a standard mercury sphygmomanometer, and the mean of these three values was used for analysis. In follow-up surveys conducted from 2011 to 2015, hypertension in this study was defined according to standard criteria as: (1) currently receiving hypertension treatment; (2) systolic blood pressure (SBP) ≥ 140 mm Hg; or (3) diastolic blood pressure (DBP) ≥ 90 mm Hg.

For PM_2.5_ exposure assessment, daily mean PM_2.5_ concentrations (µg/m^3^) for 2010–2015 were obtained for analysis (temporal resolution: 1–h automatic measurements averaged to daily means). For each participant, the examination date was used as the index date. The 0-day exposure is the daily mean concentration on the examination date. For a window k (k = 7, 15, 30, 60 days), exposure was defined as the arithmetic mean of the daily mean concentrations over the k calendar days ending on the examination date (including the examination day). The exposure windows are moving (rolling) arithmetic averages, not cumulative sums. A window average was calculated when at least 80% of the days in the window had valid measurements; otherwise the window was treated as missing, and participants with an incomplete window were excluded from the corresponding complete–case analysis.

### 2.3. Covariates

This study investigated BMI, basic, and morphological indicators provided by the database. BMI indicators included underweight, normal weight, obesity, and overweight based on the criteria from WHO. Basic indicators included age, height, and weight. Morphological indicators included arm circumference (arm_girth), waist circumference (waistline), hip circumference (hipline), and sebum thickness (sebum_thickness).

Daily activity data, encompassing intensity, frequency, and duration across various domains like household tasks, leisure, and transportation, were all derived from the International Physical Activity Questionnaire (IPAQ). The metabolic level of these activities was quantified using the metabolic equivalent (*MET*), which represents the ratio of the body’s working metabolic rate to its resting metabolic rate. In this study, activity intensity was categorized based on *MET* levels. One MET is defined as 4.2 KJ·kg^−1^·h^−1^, which is equivalent to the energy expenditure of sedentary behavior. Specific *MET* values were assigned for different activities: sleep (0.95), sedentary (1.0), work (2.0), transportation (4.0), and sports (4.0 and 8.0 *MET*/h). The total daily *MET* value for participants was calculated using the following formula:

(1) $$\mathrm{MET}=\sum \left({\mathrm{MET}}_{n}\times H_{n}\right)$$

where *MET_n_* represents the metabolic equivalent of a particular activity, and *H_n_* represents the average daily duration accordingly.

### 2.4. Model Framework

The proposed framework follows a modular three-layer architecture designed for flexibility and reusability across different exposure variables, health outcomes, and population cohorts.

Feature Engineering: This layer ingests raw environmental monitoring data and computes cumulative exposure metrics across user-defined temporal windows. In the present validation, five cumulative windows were generated as structured features, alongside standardized demographic, anthropometric, and behavioral covariates. All continuous features were standardized prior to modeling.

Multi-Model Prediction: Five complementary ML algorithms were implemented in parallel to ensure robustness across modeling paradigms. These included a general linear baseline (GLM), a regularized linear model with embedded feature selection (Lasso), a recursive partitioning method (Decision Tree), a bagging ensemble (Random Forest), and a boosting ensemble (XGBoost). The multi-model comparison strategy was designed to evaluate whether ensemble tree-based methods provided meaningful predictive gains over simpler linear approaches when multi-window exposure features were included. Detailed model specifications and hyperparameter tuning procedures are provided in Section 2.5. In this section, two distinct prediction problems are analyzed and reported separately. Hypertension risk classification: outcome = binary hypertension (yes/no); predictors = age, sex, height, weight, BMI, arm/waist/hip circumference, skinfold thickness, *MET*–weighted activity variables (sleep, work, transportation, sport, sedentary) and the five PM_2.5_ window features. SBP and DBP were excluded from the predictors of all hypertension classifiers.

Interpretability Module: A three–stage post–hoc interpretability pipeline was applied to the best-performing models: (1) permutation-based variable importance (VIP) to rank predictors by their contribution to model accuracy; (2) Shapley additive explanations (SHAP) to quantify the direction and magnitude of each feature effect on individual predictions; and (3) partial dependence plots (PDPs) to visualize marginal exposure-response relationships and identify nonlinearities and interactions. This module enables the framework to serve not only as a prediction tool but also as a hypothesis-generating instrument for exploring complex exposure-outcome dynamics.

### 2.5. Model Construction, Validation and Optimization

Continuous predictors were standardized (mean 0, SD 1) using statistics computed on the training set only. Nominal variables were encoded as indicators. To handle class imbalance (hypertension prevalence 25.3% in the analytic sample), SMOTE (synthetic minority over-sampling, 5 nearest neighbors) was applied to the standardized training data only (minority class increased from 498 to 1523 training observations); and the test set was never over-sampled. In data partitioning, participants were randomly split 80/20 at the participant level, so that all visits of one participant remained on the same side—training set: 2021 visits from 1723 participants; test set: 502 visits from 431 participants. The complete 2011 wave (873 visits) was used for training and the complete 2015 wave (1650 visits) for testing, to simulate a realistic prospective prediction scenario in year-based date split.

Five common machine learning methods were selected to construct corresponding machine learning models. The GLM establishes the relationship between the mathematical expectation of the response variable and a linear combination of predictor variables through a link function. Lasso regression is a method for fitting generalized linear models that simultaneously achieves variable selection and complexity adjustment. The decision tree algorithm is an inductive classification algorithm capable of performing both classification and regression [20]. The random forest algorithm is a classifier that uses the Bootstrap sampling method to train and predict samples [21]. XGBoost is an ensemble learning method that sorts all samples from largest to smallest (according to the first-order gradient) and traverses them to determine whether each node needs to be split [22].

For every tuned model, hyperparameters were selected by grid search with 5-fold stratified cross-validation on the training set only (optimization metric: ROC-AUC). The final model was refitted on the full training set and evaluated once on the held-out test set. Search spaces and final parameters were set as GLM—unregularized logistic regression (L2 penalty, C = 10^6^); Lasso—penalty C ∈ {10^(−3.5…0.5), 20 points}, final C = 0.739; Decision Tree—max_depth ∈ {3,5,8,12}, min_samples_leaf ∈ {2,5,10,20}, final (8, 20); Random Forest—mtry ∈ {4,8,12,16}, min_samples_leaf ∈ {2,5}, 300 trees for tuning and 600 for the final model, final (mtry 4, leaf 2, 600 trees); XGBoost—max_depth ∈ {3,5,7}, learning_rate ∈ {0.02,0.05,0.1}, subsample 0.8, colsample_bytree 0.8, 300 trees for tuning and 600 for the final model, final (depth 7, learning rate 0.05, 600 trees). The model performance was evaluated on the held-out test sets with the area under the receiver operating characteristic curve (AUC, with 95% bootstrap confidence intervals from 2000 resamples), accuracy, sensitivity, specificity, precision, F1-score, the Brier score, calibration intercept/slope, and a decision-curve analysis. Pairwise AUC comparisons between models were tested with a paired bootstrap test.

### 2.6. Statistical Analysis

Descriptive statistics compared hypertensive and non-hypertensive participant-visits with *t*-tests (Welch) for continuous variables and chi-square tests for categorical variables. Pearson correlation was used only for pairs of continuous variables; associations of binary variables with SBP/DBP/hypertension were quantified with point-biserial correlation, and multi-category factors were tested with one-way ANOVA (continuous outcomes) or the chi-square test (binary outcome). Correlation figures include continuous variables only.

Logistic regression was used for the hypertension classifiers and explanatory analyses. We used window-specific exposure-response models to relate each PM_2.5_ window (entered separately, per 10 µg/m^3^) to hypertension, SBP, and DBP, adjusting for age, sex, BMI, and total *MET*-activity. Formal interaction (effect-modification) tests were conducted using product terms (7-day and 60-day PM_2.5_ × total MET, ×sleep duration, ×BMI, ×sex) in adjusted logistic models, with Wald tests and Benjamini–Hochberg false-discovery-rate (FDR) control applied across the eight interaction tests. Ablation analyses compared each algorithm with and without the five PM_2.5_ window features using a paired bootstrap test. The interaction tests were corrected using the Benjamini- Hochberg false-discovery rate. Window-specific exposure-response estimates and model comparisons are reported with 95% confidence intervals and interpreted as effect sizes. The remaining descriptive, importance-related, and interpretability-related outputs are exploratory and were not adjusted for multiplicity. Variance inflation factors were computed for the multi-window logistic model to quantify collinearity among the nested windows. To examine the hypertension risk classification (binary outcome) with various categorical and continuous variables, five machine learning models were constructed: Generalized Linear Model (GLM), Lasso, Decision Tree, Random Forest (RF), and XGBoost. Table 2 specifies the outcome, predictor set, validation scheme, and evaluation metrics for every analysis.

Following 1000 resampling iterations and standardization, a standardized regression coefficient-variation decomposition model was developed. Numerical variables associated hypertension were categorized into air pollution, daily activity, basic morphology, body indicators, and BMI. The contribution of independent variables to the dependent variable in the regression model was compared based on their numerical characteristics. The standardized regression coefficient’s magnitude indicates the effect size of each variable on a unit standard deviation change of the dependent variable, allowing for the determination of independent variables’ importance by comparing these magnitudes. All analyses were performed in R 4.4 (R Core Team). Statistical significance was set at *p* < 0.05 (two-sided), and effect sizes are reported with 95% confidence intervals (CIs). The machine learning models in this study were based on classification regression and implemented using the “tidymodels” series packages in R. Variable importance was determined using the “VIP” package. SHAP plots and PDP analysis were determined using the “shapviz” and “pdp” packages, respectively. SMOTE was used for imbalanced data processing using the “themis” package in R. Five machine learning R packages—“stats::glm” (GLM), “ranger” (RF), “xgboost,glmnet” (Lasso), and “rpart” (Decision Tree)—were used for model analysis.

## 3. Results

### 3.1. Validation Cohort Characteristics

The analytic sample comprised 2523 participant visits from 2154 unique Beijing CHNS participants (873 visits from the 2011 wave and 1650 from the 2015 wave). Of these, 639 visits (25.3%) met the hypertension definition.

Table 3 compares the characteristics of hypertensive and non-hypertensive participant visits. Hypertensive participants were, on average, approximately 10 years older (57.7 ± 12.5 vs. 48.0 ± 15.1 years), had a higher BMI (26.3 ± 3.5 vs. 24.3 ± 3.8 kg/m^2^), and were more frequently male (53.5% vs. 44.4%). They also exhibited higher body weight, waist/hip/arm circumference, and skinfold thickness, as well as lower total and work-related physical activity (all *p* < 0.001, except skinfold thickness, *p* = 0.006). Mean PM_2.5_ exposures was lower in the hypertensive group across all exposure windows (0-day: 87.2 vs. 100.0 µg/m^3^; 7-day: 89.2 vs. 100.0 µg^3^m^3^; 60-day: 69.5 vs. 79.1 µ^3^/m^3^; Welch *p* < 0.001 for each window).

Among continuous variables, the mean PM_2.5_ concentration was 100.0 ± 87.4 μg/m^3^ in the normotensive group and 87.2 ± 80.1 μg/m^3^ in the hypertensive group, with the hypertensive group exhibiting significantly lower PM_2.5_ levels (*p* < 0.001). The mean age for the normotensive group was 48.0 ± 15.1 years, compared to 57.7 ± 12.5 years for the hypertensive group, indicating that the hypertensive group was significantly taller (*p* < 0.001). BMI averaged 24.3 ± 3.8 kg/m^2^ in the normotensive group and 26.3 ± 3.5 kg/m^2^ in the hypertensive group. The hypertensive group had a significantly larger BMI (*p* < 0.001) associated with an increased risk of hypertension. Arm, waist and hip circumferences were 29.0 ± 5.2, 84.1 ± 11.5 and 95.8 ± 9.9 mm in the normotensive group and 29.8 ± 4.2, 89.8 ± 12.1 and 98.8 ± 10.0 mm in the hypertensive group. The hypertensive group showed significantly greater arm, waist and hip circumferences (*p* < 0.001), indicating that they were associated with an increased risk of hypertension. Sit and work levels were 6.7 ± 5.4 and 6.1 ± 10.3 in the normotensive group and 5.8 ± 5.3 and 3.8 ± 9.5 in the hypertensive group. The hypertensive group exhibited significantly lower physical activity levels (*p* < 0.001), suggesting that lower physical activity was associated with a reduced risk of hypertension. In summary, age, weight, BMI, arm circumference, waist circumference, hip circumference, sit and work were identified as statistically significant factors associated with hypertension (*p* < 0.001).

### 3.2. Correlation Analysis

In this study, the Pearson correlation coefficient method was used to explore the relationships between SBP, DBP, and various variables, including age, height, body weight, sleep duration, working, transportation activities, sports, sedentary activities, body mass index, and categorized PM_2.5_ concentrations.

**Figure 1:**
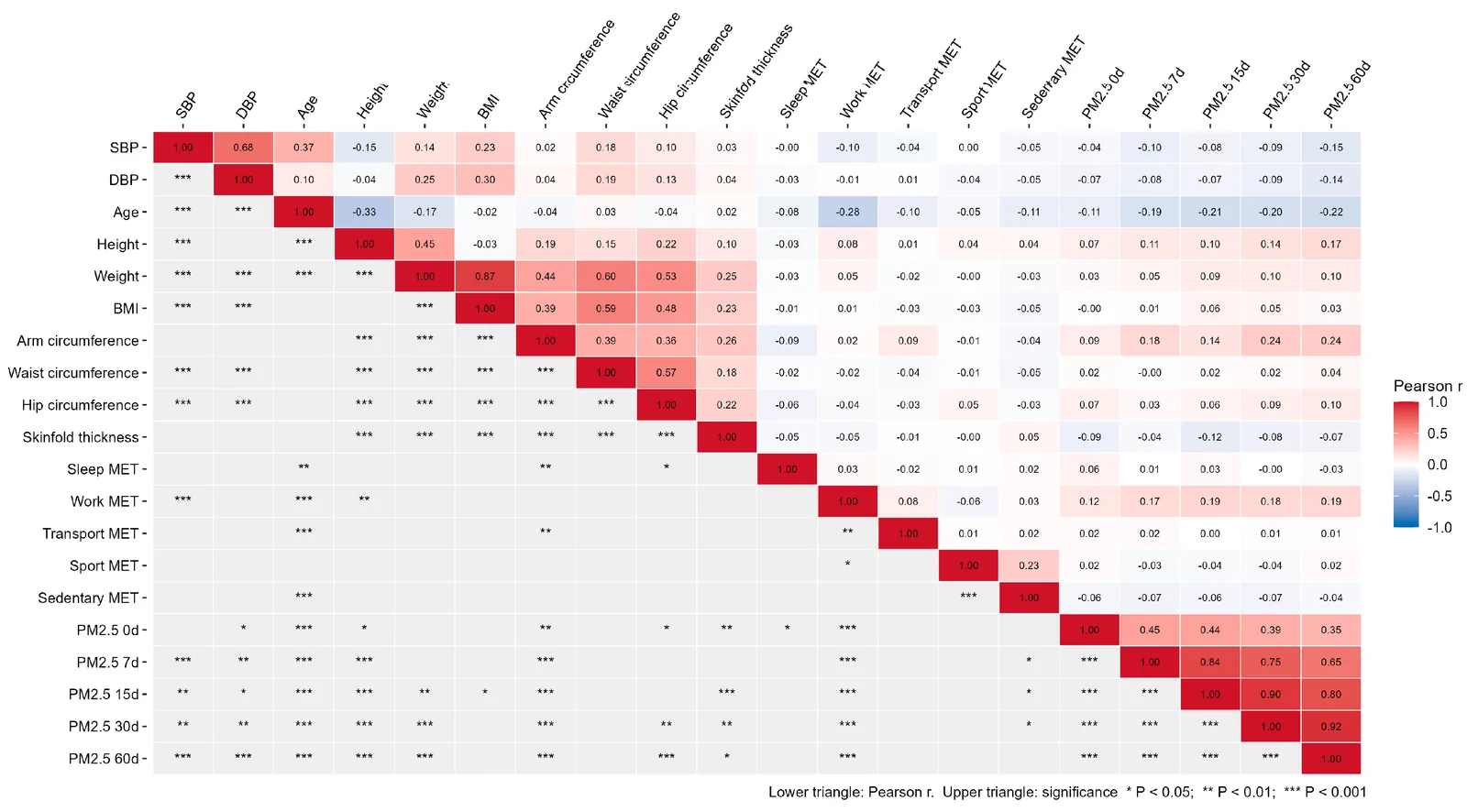
Pearson correlation analysis among BP and all numeric variables for male residents.

Figure 1 illustrates the associations of SBP and DBP with various variables among males. Among continuous variables, age exhibited a highly significant positive correlation with both SBP and DBP (r = 0.37 and r = 0.10, *p* < 0.001). Height was significantly negatively correlated with SBP (r = −0.15, *p* < 0.001, but no significant correlation was with DBP. BMI was significantly and positively correlated with SBP and DBP (r = 0.23 and r = 0.30, *p* < 0.001). Body weight showed a highly significant positive correlation with SBP and DBP (r = 0.14 and r = 0.25, *p* < 0.001). Age was highly significantly positively correlated with both SBP and DBP (r = 0.37 and r = 0.10, *p* < 0.001). Waist circumference was highly significantly positively correlated with SBP and DBP (r = 0.18 and r = 0.19, *p* < 0.001). Hip circumference exhibited a highly significant positive correlation with SBP and DBP (r = 0.10 and r = 0.13, *p* < 0.001). For categorical variables, work was significantly negatively correlated with SBP (r = −0.10, *p* < 0.001, but no significant correlation was with DBP. Sleep, transportation, sport and sedentary behavior presented no significant correlation with SBP and DBP. PM_0_ day was not associated with SBP but was negatively correlated with DBP (r = −0.07, *p* < 0.05). PM-7 day showed a highly significant positive correlation with both SBP and DBP (r = −0.10, *p* < 0.001 and r = −0.08, *p* < 0.01). PM-15 day had a highly significant negative correlation with SBP (r = −0.08, *p* < 0.01) and a significant negative correlation with DBP (r = −0.07, *p* < 0.05). PM-30 day was highly significantly negatively correlated with SBP and DBP (r = −0.09 and r= −0.09, *p* < 0.01). PM-60 day exhibited a highly significant negative correlation with both SBP and DBP (r= −0.15 and r= −0.14, *p* < 0.001).

**Figure 2:**
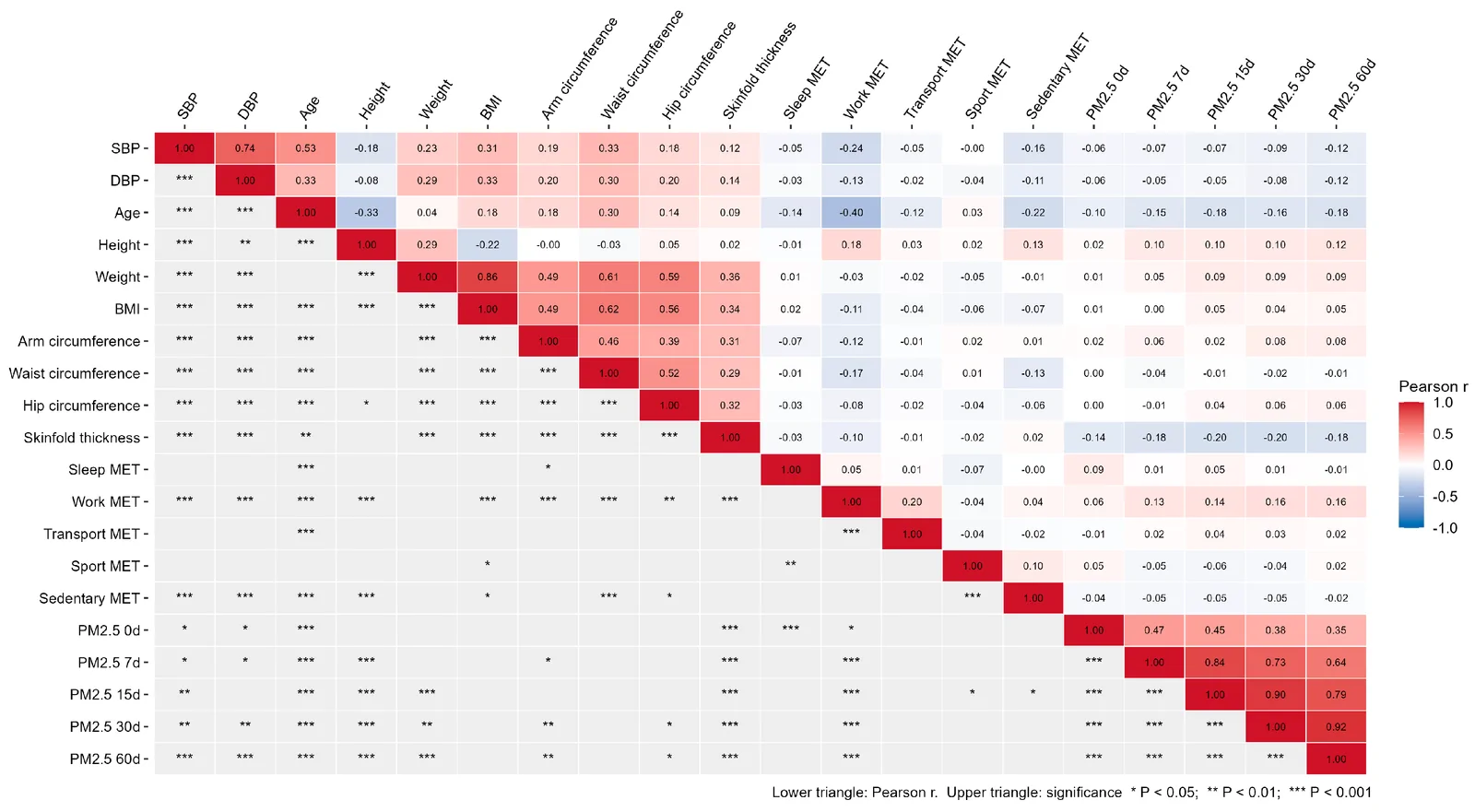
Pearson correlation analysis among BP and all numeric variables for female residents.

Figure 2 illustrates the associations between SBP and DBP and various variables in females. For continuous variables, SBP showed an extremely significant correlation with age (r = 0.53, *p* < 0.001), height (r = −0.18, *p* < 0.001), body weight (r = 0.23, *p* < 0.001), BMI (r = 0.31, *p* < 0.001), arm circumference (r = 0.19, *p* < 0.001), waist circumference (r = 0.33, *p* < 0.001), hip circumference (r = 0.18, *p* < 0.001), and skinfold thickness (r = 0.12, *p* < 0.001). DBP was extremely significantly correlated with age (r = 0.33, *p* < 0.001), body weight (r = 0.29, *p* < 0.001), BMI (r = 0.33, *p* < 0.001), arm circumference (r = 0.20, *p* < 0.001), waist circumference (r = 0.30, *p* < 0.001), hip circumference (r = 0.20, *p* < 0.001), and skinfold thickness (r = 0.14, *p* < 0.001). It also exhibited an extremely significant negative correlation with work (r = −0.13, *p* < 0.001) and sedentary behavior (r = −0.11, *p* < 0.001). Among categorical variables, SBP was extremely significantly negatively correlated with work-related activity (r = −0.24, *p* < 0.001) and sedentary behavior (r = −0.16, *p* < 0.001). No significant correlation was observed with sleep, transportation or sports. Furthermore, SBP showed a significant negative correlation with PM-0 day (r = −0.06, *p* < 0.01) and PM-7 day (r = −0.07, *p* < 0.05), and an extremely significant negative correlation with PM-15 day (r = −0.07, *p* < 0.01), PM-30 day (r = −0.08, *p* < 0.01), and PM-60 day (r = −0.12, *p* < 0.001). DBP also showed a negative correlation with PM-0 day (r = −0.06, *p* < 0.05), PM-7 day (r = −0.05, *p* < 0.05), PM-30 day (r = −0.08, *p* < 0.01), and an extremely significant negative correlation with PM-60 day (r = −0.12, *p* < 0.001).

The correlation analysis results reveal sex-specific associations of SBP and DBP with anthropometric, behavioral, and environmental factors. In males, age, BMI, body weight, waist and hip circumference were positively associated with both SBP and DBP, whereas height and work were inversely associated with SBP. In females, age, body weight, BMI, arm, waist, hip circumference, and skinfold thickness were positively associated with both pressures; height was inversely associated with SBP, and work-related activity and sedentary behavior were inversely associated with SBP and DBP. PM_2.5_ exposures generally showed negative associations, especially PM_2.5_ 60d. Overall, age and adiposity are positive correlates, while PM_2.5_ exposures and some behaviors are inverse correlates, with clear sex differences. Because the five exposure windows are nested, their inter-correlation and collinearity were also quantified (Table S2). Pearson correlations among the windows ranged from r = 0.35 (0-day vs. 60-day) to r = 0.92 (30-day vs. 60-day). In the joint multi-window logistic model, the variance inflation factors were 1.3 (0-day), 3.5 (7-day), 8.0 (15-day), 13.0 (30-day), and 7.0 (60-day).

### 3.3. Model Performance and Comparative Evaluation

**Figure 3:**
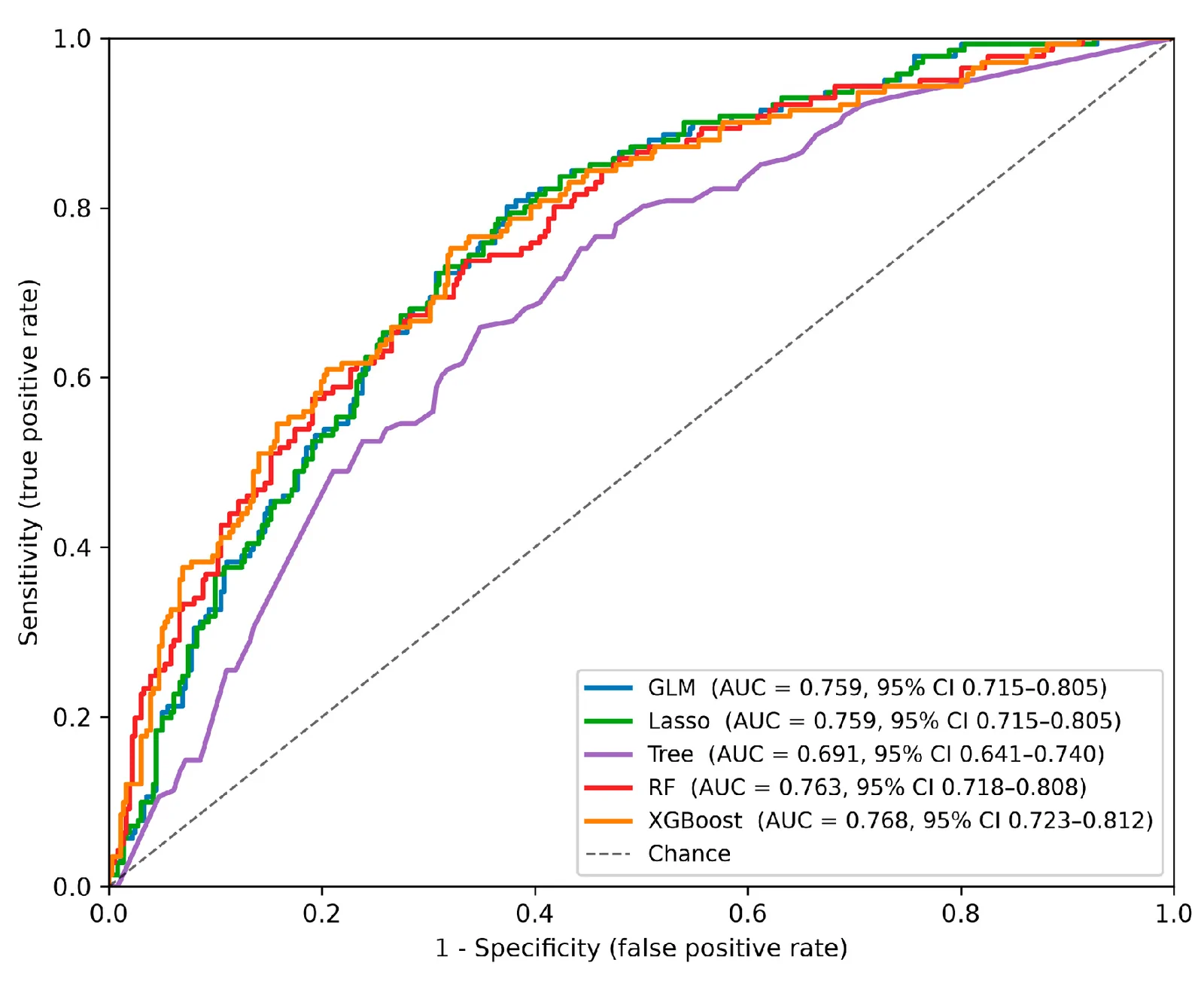
Machine learning models predict performance.

After excluding SBP/DBP from the predictors and using the person-level split, the five models were trained with SMOTE-balanced training data and evaluated on the held-out test set of 502 visits. This study employed cross-validation and other methods to evaluate and compare models, primarily using the receiver operating characteristic (ROC) curve to assess performance. The ROC curve illustrates the balance between sensitivity and specificity. The X-axis represents specificity (false positive rate), with values closer to zero indicating higher accuracy. The Y-axis denotes sensitivity (true positive rate), where larger values signify better accuracy. The Area Under the ROC Curve (AUC) quantifies predictive accuracy; a higher AUC, corresponding to a larger area under the curve, indicates superior performance. A curve positioned closer to the top-left corner (lower X, higher Y) suggests better predictive capability. Figure 3 displays the five machine learning models. Cross-validation results showed that all ROC curves were close to the (0,1) coordinate point, and all five models achieved relatively high AUC values. These results confirm that the models possess high predictive accuracy and favorable classification performance for factors associated with hypertension. Notably, the XGBoost and RF models outperformed GLM, Lasso, and Tree.

**Figure 4:**
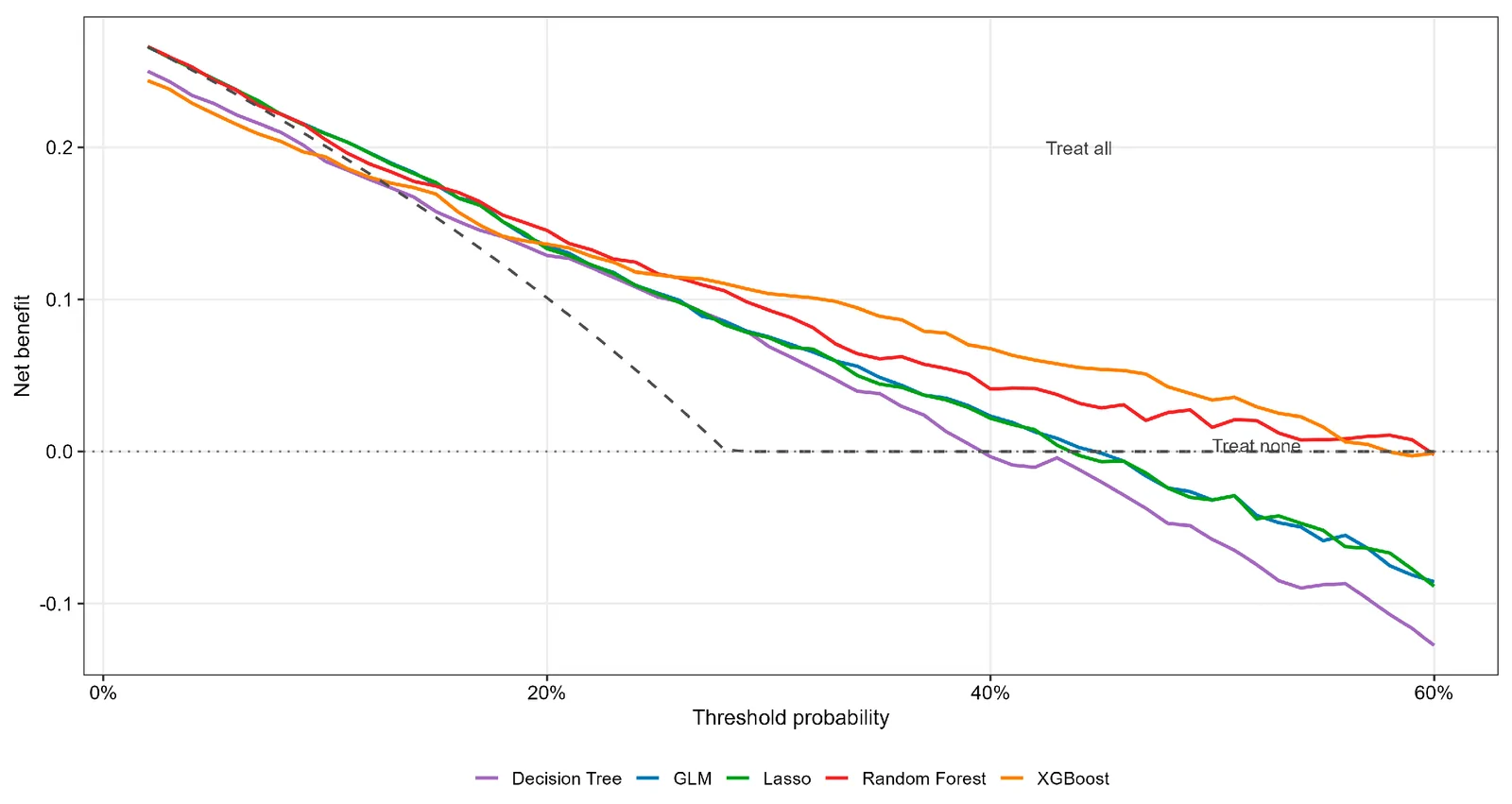
Decision-curve analysis of the five hypertension classifiers.

Table 4 reports the performance metrics. XGBoost achieved the highest test AUC (0.768, 95% CI 0.723–0.813), followed by RF (0.763, 95% CI 0.718–0.808), Lasso (0.759), and GLM (0.759). The Decision Tree performed clearly lower (0.692, 95% CI 0.642–0.740). Paired tests comparing models against XGBoost (the best model) showed no significant difference for RF (ΔAUC −0.005, *p* = 0.63), GLM (ΔAUC −0.009, *p* = 0.64), or Lasso (ΔAUC −0.008, *p* = 0.65) (Table S1). However, the Decision Tree was significantly worse (ΔAUC −0.076, *p* < 0.001). XGBoost did not clearly outperform RF because, in this moderate-size, low-dimensional problem, the boosting ensemble extracts little additional signal beyond what bagged trees already capture; their test CIs overlap, and the paired difference is not significant. When models were trained on the complete 2011 wave (873 visits) and tested on the complete 2015 wave (1650 visits), test AUCs were 0.720 (95% CI 0.695–0.743) for GLM, 0.718 (95% CI 0.692–0.744) for RF, and 0.678 (95% CI 0.650–0.705) for XGBoost. In the decision-curve analysis (Figure 4), the three main models provided a higher net benefit than either ‘treat all’ or ‘treat none’ across threshold probabilities of approximately 0.15–0.45.

### 3.4. Feature Importance and Interpretability Analysis

**Figure 5:**
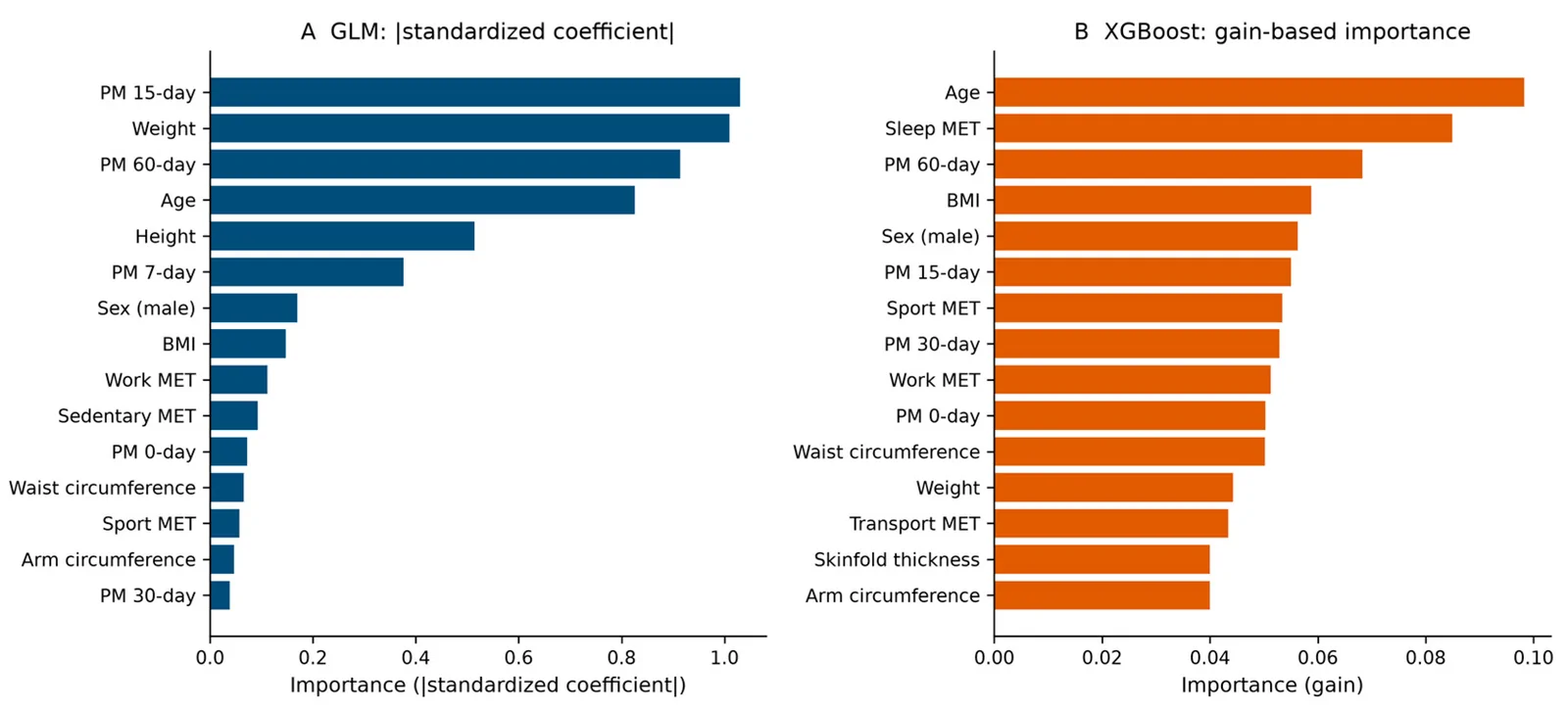
The importance variables of the GLM and XGBoost models for hypertension.

A GLM model was constructed following variable standardization (Figure 5A), with hypertension as the dependent variable. The GLM revealed PM_2.5_ (15-day average) as the most critical factor associated with hypertension, indicating an increased risk with higher PM_2.5_ concentrations. Body weight ranked second, suggesting a link between body fat accumulation and hypertension. PM_2.5_ (60-day average) ranked third in contribution to the model factor, implying that prolonged PM_2.5_ exposure may affect the onset and progression of hypertension. Age also played an important role in relation to hypertension, while other variables showed relatively low importance. AnXGBoost model was also developed, after variable standardization, to predict the factors associated with hypertension. Figure 5B illustrates the prediction results from the XGBoost model. The variable importance ranking was: age > sleep > PM_2.5_ (60-day exposure) > BMI > sex > PM_2.5_ (15-day exposure) > sports > PM_2.5_ (30-day exposure) > work > PM_2.5_ (0-day exposure) > waist circumference> body weight > transportation activity > skinfold thickness > arm circumference. Among these, age, sleep, and PM_2.5_ (60-day exposure) demonstrated higher importance.

**Figure 6:**
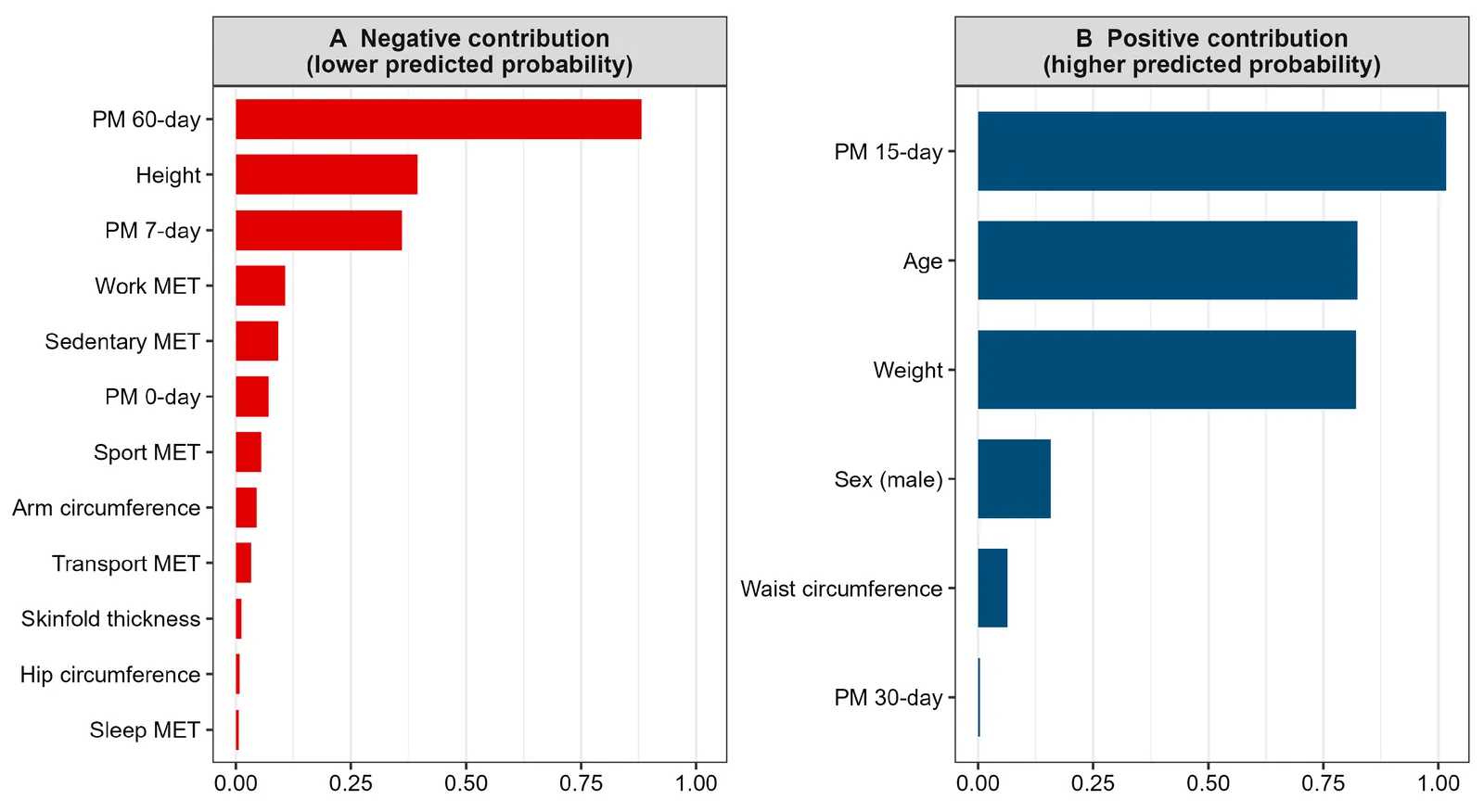
The importance variables of the Lasso model for hypertension.

A Lasso regression model, which was built with standardized variables, identified factors associated with hypertension (Figure 6). The model revealed several factors with an inverse effect on hypertension, ranked by importance: PM_2.5_ (60-day exposure) > height > PM_2.5_ (7-day exposure) > work > sedentary behavior > PM_2.5_ (0-day exposure) > sport > arm circumference > transport > skinfold thickness > hip circumference > sleeping. Conversely, the following variables showed a positive effect on hypertension, ranked by importance: PM_2.5_ (15-day exposure) > age > weight > sex > waist circumference > PM_2.5_ (30-day exposure). Categorical variables demonstrated significant and varied effects on hypertension. For BMI categories, height, arm circumference, hip circumference and skinfold thickness had inverse effects, while normal weight and waist circumference showed a positive effect. Among physical activity categories, all the activities were negatively associated with hypertension. Different PM_2.5_ lag exposure indicators also showed distinct directions of association: PM_2.5_ (0-day, 7-day and 60-day exposure) showed a negative effect, and PM_2.5_ (15-day exposure) presented a positive effect.

**Figure 7:**
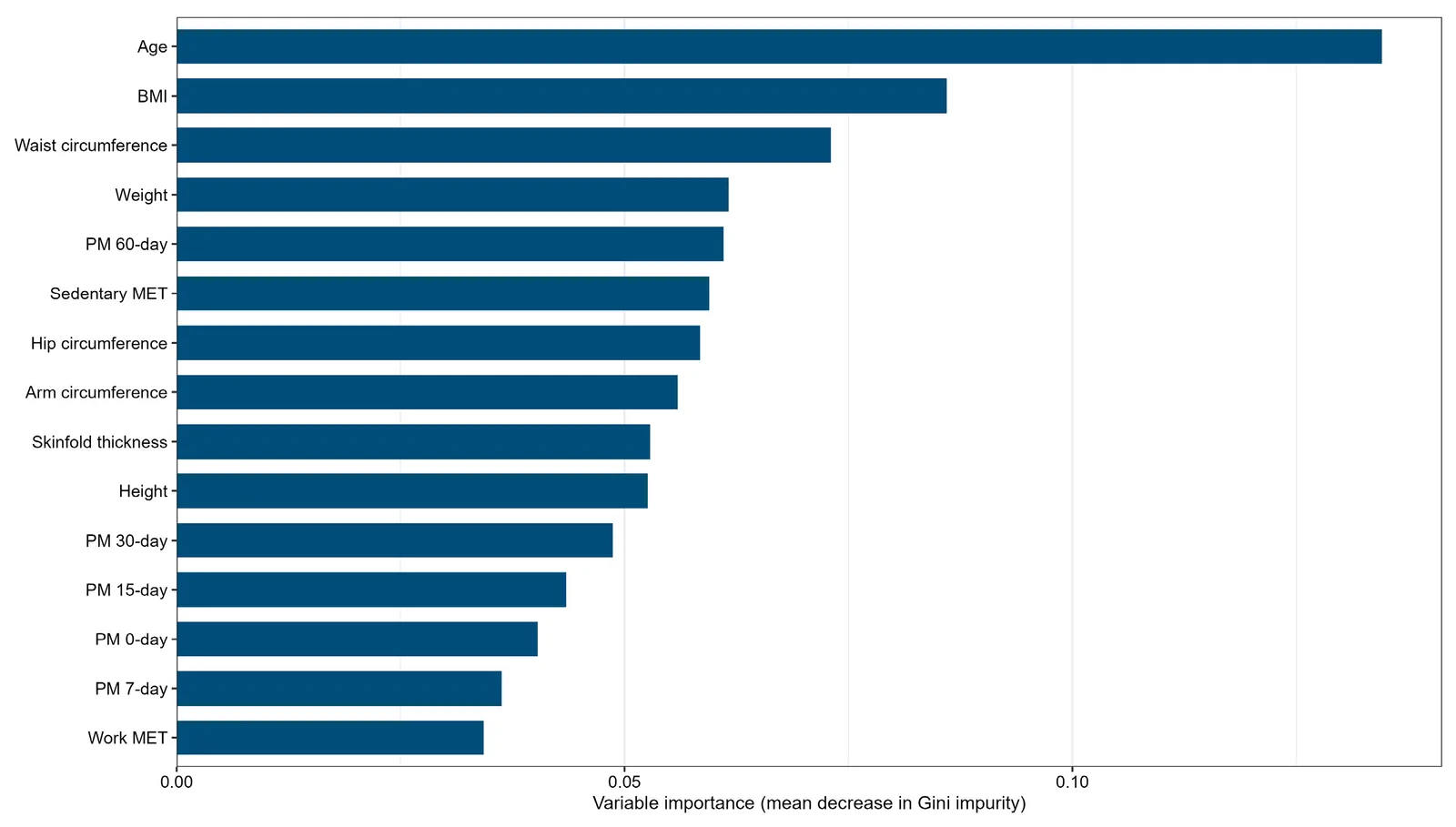
Variable importance of the random forest classification model for hypertension.

**Figure 8:**
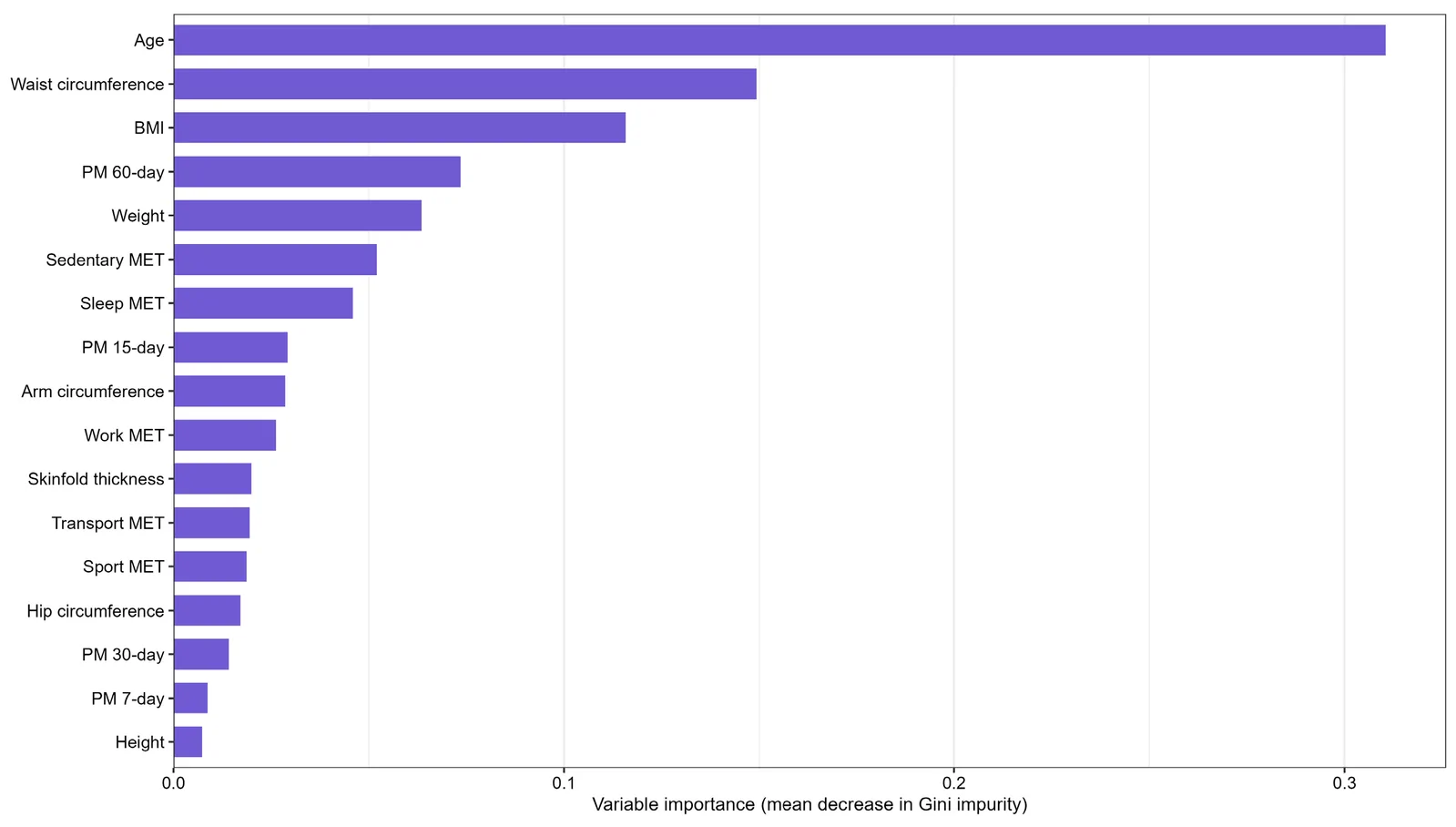
Variable importance of the decision tree classification model for hypertension.

Figure 7 and Figure 8 show the impurity-based variable importance of the random forest and decision tree classifiers for hypertension, where the outcome was hypertension status rather than a continuous blood pressure measure. Age was by far the most important predictor (mean decrease in Gini 0.135), followed by BMI (0.086) and waist circumference (0.073). Body weight (0.062) and the 60-day PM_2.5_ window (0.061) ranked fourth and fifth. Among the five exposure windows, the 60-day window was the most important, while the 7-day window ranked lowest (0.036). The intermediate windows (30-, 15-, and 0-day) occupied middle positions (0.049, 0.043, and 0.040). The prominence of age, BMI, and central adiposity measures aligns with established epidemiological knowledge, providing an internal validation of the corrected pipeline. The dominance of the longer PM_2.5_ window over shorter windows indicates that the multi-window exposure features carried most of their (modest) predictive information at a longer timescale. Since impurity-based importance measures the average reduction in node impurity and does not encode the direction of an association, and because the nested exposure windows are strongly correlated, these values are interpreted solely as a relative ranking of predictive contribution. The direction and magnitude of each window were therefore quantified separately in the single-window adjusted models and cross-checked with SHAP, partial dependence, and ablation analyses.

### 3.5. SHAP and PDP Analysis

**Figure 9:**
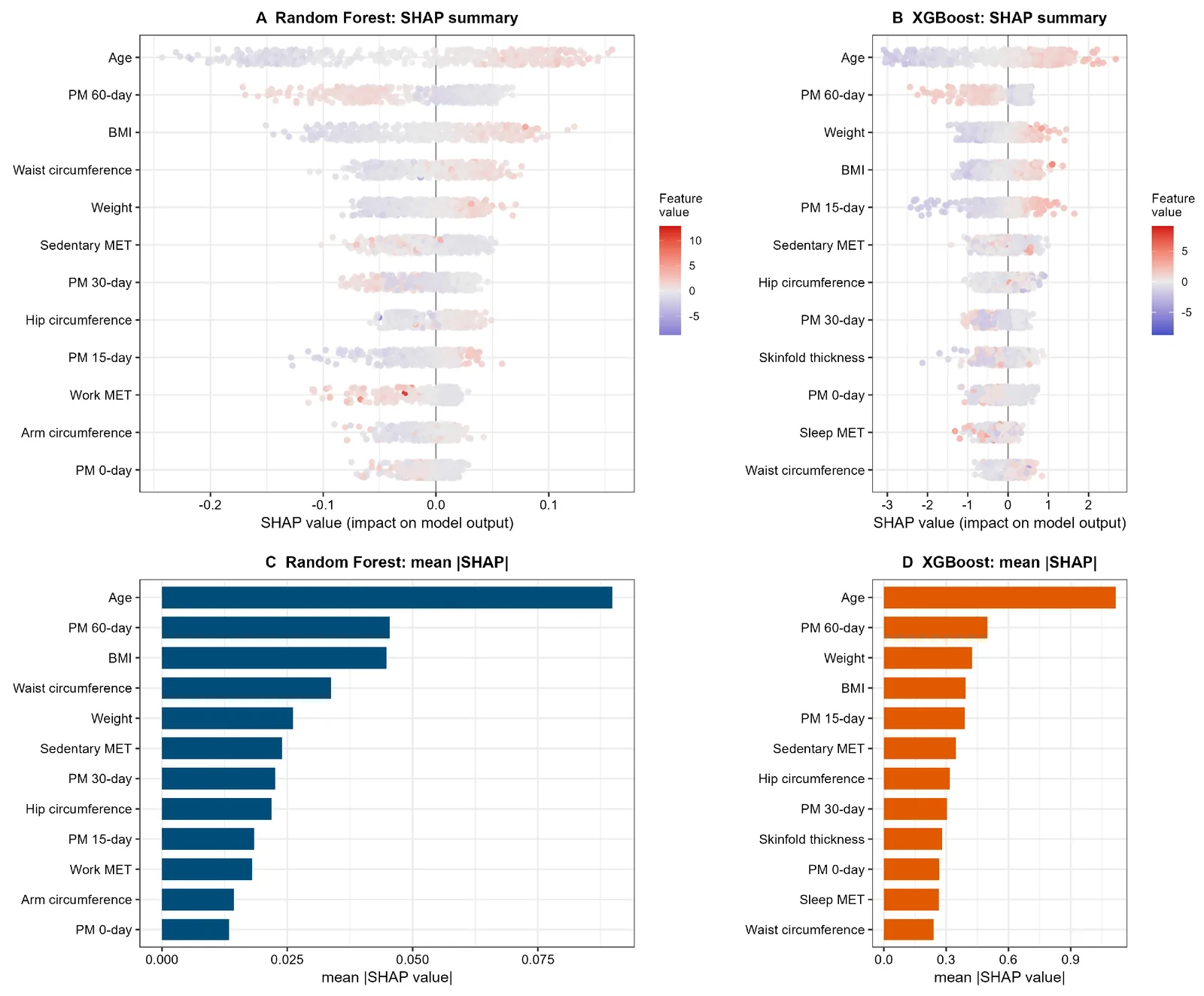
SHAP analysis of the corrected hypertension classifiers on the test set. (**A**,**B**) SHAP summary (beeswarm) plots for Random Forest (**A**) and XGBoost (**B**); (**C**,**D**) mean |SHAP| importance for Random Forest (**C**) and XGBoost (**D**). Points are colored by the standardized feature value (blue = low, red = high); SHAP values are in units of log-odds contribution to the predicted probability of hypertension.

**Figure 10:**
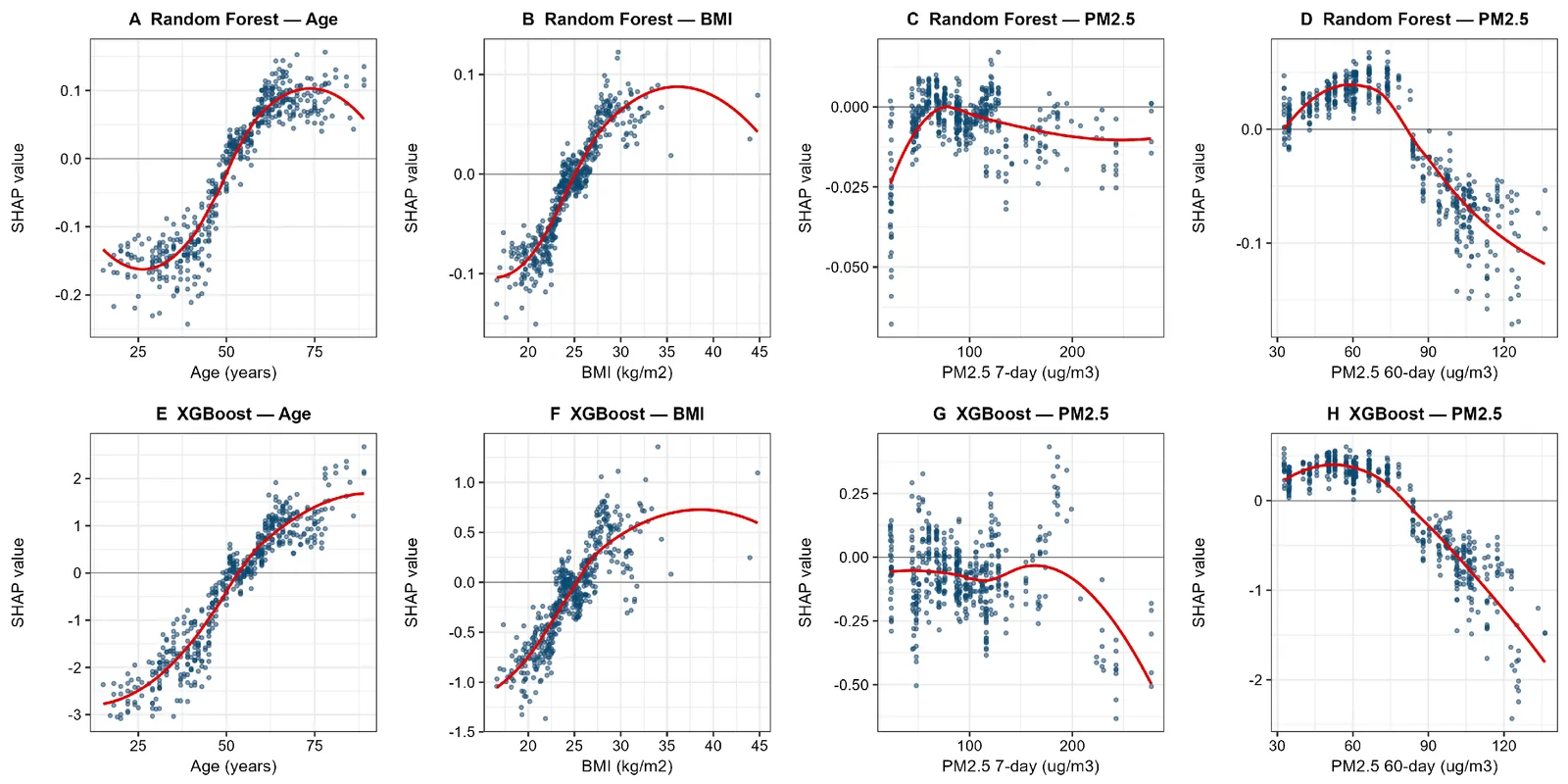
SHAP dependence plots for age, BMI, the 7-day and the 60-day PM_2.5_ windows for Random Forest (**top**) and XGBoost (**bottom**).

**Figure 11:**
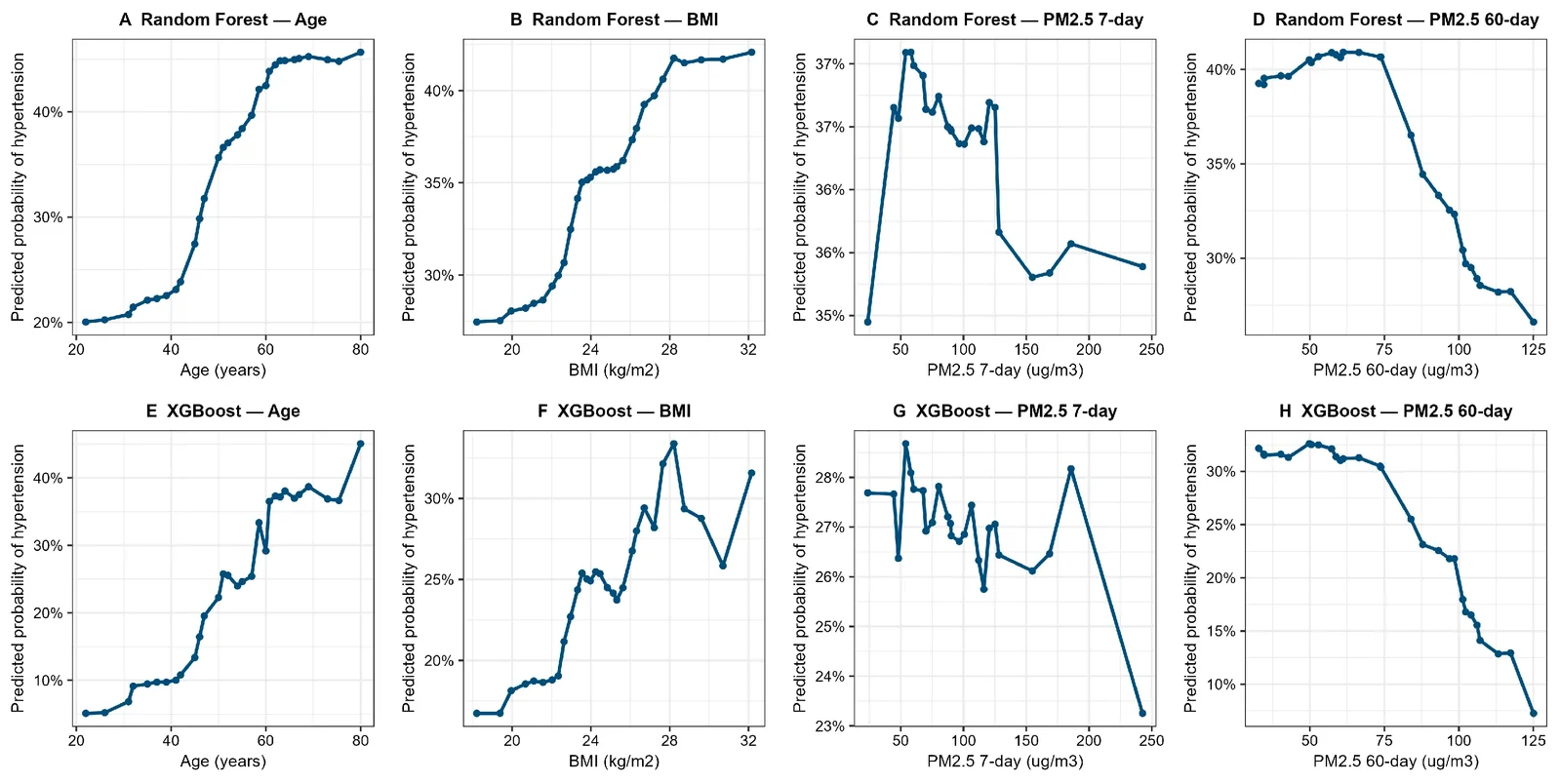
PDP for age, BMI, the 7-day and the 60-day PM_2.5_ windows for Random Forest (**top**) and XGBoost (**bottom**).

Due to the intercorrelation and collinearity of the five nested exposure windows, coefficients and importance values of individual windows in the joint model are unstable and should not be interpreted as independent effects. SHAP dependence and PDP curves for a given window are computed conditionally on the other windows; therefore, their shapes can be attenuated or even reversed relative to the marginal association. We thus use SHAP/PDP to describe the shape and relative relevance of contributions within the joint model. RF and XGBoost models showed relatively higher AUCs, which were then used to conduct the SHAP analysis. Figure 9, Figure 10 and Figure 11 present various analyses of RF and XGBoost models evaluated on the test set. Figure 9 displays SHAP summary (beeswarm) plots and mean |SHAP| importance for Random Forest (A, C) and XGBoost (B, D). Age is the dominant SHAP contributor in both models, with a mean |SHAP| of 0.090 in RF and the largest contribution in XGBoost. Among the PM_2.5_ windows, the 60-day window is the largest contributor, ranking second only to age in XGBoost, which aligns with the consensus ranking. Figure 10 presents SHAP dependence plots for age, BMI, and the 7-day and 60-day PM_2.5_ windows. The dependence plots show a monotonically decreasing contribution across the range of the 60-day window. Figure 11 illustrates partial dependence plots for age, BMI, and the 7-day and 60-day windows for RF and XGBoost. The predicted probability of hypertension increases with age, steeply up to approximately 60 years and then flattening, and also increases with BMI. Conversely, it decreases across the range of the 60-day PM_2.5_ window, while the 7-day window exhibits a flat marginal profile.

Both interpretability outputs indicate that age is the strongest predictor, exhibiting a consistent, non-linear positive pattern. In the PDPs, the predicted probability of hypertension rises steeply with age, flattening after approximately age 60. This probability increases from about 20% to 46% across the observed age range in the RF model and from roughly 5% to 45% in XGBoost. Correspondingly, the SHAP dependence plots shift from negative SHAP values at younger ages to positive values at older ages (mean SHAP −0.15 to +0.09 in RF; −2.04 to +1.15 in XGBoost when comparing the lowest and highest quartiles), confirming that age increasingly contributes to the predicted probability of hypertension. BMI demonstrates an approximately monotonic positive relationship in both models. The predicted probability of hypertension increases by roughly 15 percentage points across the observed BMI range (about 18–32 kg/m^2^) in both RF (27% → 42%) and XGBoost (17% → 32%). Similarly, the SHAP dependence plots transition from negative to positive values (RF: −0.08 to +0.06; XGBoost: −0.73 to +0.44 across quartiles). The shape of this relationship is nearly linear and fully consistent with the well-established epidemiological association between adiposity and hypertension. 

The short-term exposure window contributes weakly and without a clear direction in the joint models. The PDP is essentially flat in RF and only slightly decreasing in XGBoost (28% → 23%). Likewise, the SHAP dependence plots are almost horizontal with very small magnitudes and wide individual scatter. Given the strong correlation among the five windows, this flat profile in the joint model should not be interpreted as the causal effect of the 7-day window. The longer-term window is the dominant exposure feature, exhibiting a clear inverse marginal relationship in both models. The predicted probability of hypertension decreases monotonically with increasing 60-day exposure, from approximately 39% to 27% in RF and more steeply from 32% to 7% in XGBoost. The SHAP dependence plots mirror this pattern, with SHAP values shifting from positive at low exposure to strongly negative at high exposure.

**Figure 12:**
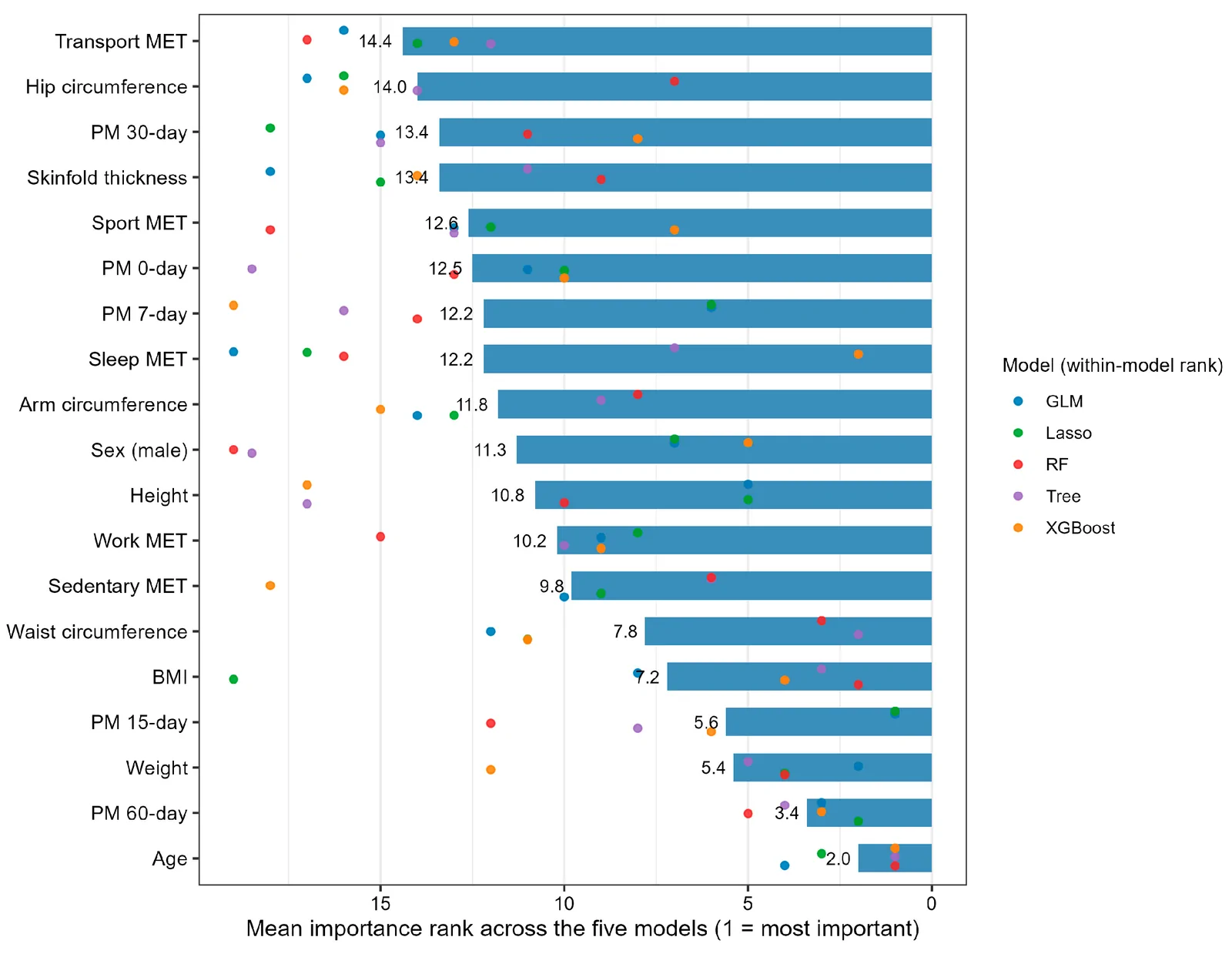
Consensus (integrated) feature-importance ranking across the five models.

Because individual models rank variables differently, we derived a consensus ranking by averaging each variable’s within-model importance rank across the five models (Figure 12) (Absolute standardized coefficients were used for GLM/Lasso, impurity importance for Tree/RF, and gain importance for XGBoost). For each predictor, its within-model importance rank (1 = most important) is averaged over the five models; and variables are ordered by the mean rank. Age was the most important predictor in every model (mean rank 2.0), followed by the PM_2.5_ (60-day exposure) window (3.4), body weight (5.4), the PM_2.5_ (15-day exposure) (5.6), and BMI (7.2). Waist circumference ranked highest among the anthropometric variables in the tree models. Among the PM_2.5_ windows, the 60-day window was consistently the most important, while the 7-day window was the least important and most unstable across models.

In summary, the SHAP dependence (Figure 10) and partial dependenceplots (Figure 11) are mutually consistent, with agreement between RF and XGBoost for all four predictors. Age and BMI show positive effects, PM_2.5_ in the 60-day window shows a negative effect, and PM_2.5_ in the 7-day window is essentially neutral in the joint multi-window models. These marginal and instance-level outputs complement the global importance ranking (Figure 12). Additionally, due to strong correlations between nested windows, each window was also analyzed separately in adjusted models (Table 5). All windows showed inverse associations with hypertension, which became monotonically stronger for longer windows. Formal interaction tests revealed that only the PM_2.5_ (60-day exposure) × BMI interaction was statistically significant after Benjamini–Hochberg FDR control, indicating that the inverse 60-day association was stronger at higher BMI.

## 4. Discussion

This study developed and validated an interpretable machine learning framework that integrates multi-window environmental exposure features for hypertension risk assessment on a well-defined analytic sample of 2523 participant-visits from 2154 unique Beijing CHNS participants. After analyzing at the participant level with person-level and year-based splits, all five algorithms showed realistic discrimination (test AUC 0.69–0.77), with age, BMI and waist circumference consistently ranked among the most important predictors [23,24,25,26,27]. The 60-day PM_2.5_ window was the most important exposure feature, and the five PM_2.5_ windows added a small but statistically significant predictive gain in the ensemble models (ablation ΔAUC ≈ 0.03, *p* = 0.03–0.05).

Several lines of evidence support the reliability of the analytical results and the applicability of the machine learning models employed in this framework. First, all five ML algorithms—spanning linear, regularized, tree-based, and ensemble paradigms—converged on consistent predictor rankings, with age, BMI, and waist circumference identified as the most important features across every model. This cross-algorithm concordance substantially reduces the likelihood that findings are artifacts of any single modeling approach. Second, the prominence of established cardiovascular risk factors in the feature importance rankings serves as an internal validation: the framework correctly recovers well-documented epidemiological relationships without requiring their a priori specification, demonstrating that the data and modeling pipeline are capable of capturing genuine risk signals. Third, all models were evaluated using rigorous cross-validation procedures with standardized 80/20 train-test splits, and performance was assessed via ROC-AUC metrics that account for both sensitivity and specificity. The consistently high AUC values across all five algorithms indicate that the models generalize beyond the training data rather than overfitting to noise. Fourth, the contribution of multi-window PM_2.5_ features, although smaller in magnitude than classical risk factors, was statistically significant and reproducible across multiple model specifications. Finally, the direction and magnitude of identified associations are broadly consistent with prior epidemiological and panel studies, further corroborating the biological plausibility of the model outputs. Collectively, these factors provide confidence in the reliability of the framework results and support its applicability for hypertension risk assessment, even in the absence of an ablation-based feature removal experiment.

A defining feature of the proposed framework is its systematic incorporation of cumulative environmental exposure across multiple temporal windows. Prior ML-based hypertension prediction models have predominantly treated air pollution as a single aggregate measure—typically an annual average—or excluded it entirely [28,29,30]. At the same time, the nested windows are strongly correlated, so per-window importance, SHAP, and PDP values from the joint model must be interpreted cautiously. We therefore pre-analyzed window-specific single-window models as the recommended basis for direction and magnitude, and a consensus importance ranking (Figure 11) as the basis for relative relevance. Under this protocol, the 60-day window consistently showed the strongest association, and the 7-day window the weakest and least stable contribution.

BMI indicators exhibited significant modifying effects on the PM_2.5_–hypertension relationship, consistent with evidence that behavioral factors modulate susceptibility to environmental exposures [17,31,32,33,34]. The interpretability module revealed the interactions without requiring a priori specification, illustrating the value of ML-based approaches for hypothesis generation in complex exposure-outcome settings. This framework improves upon earlier concatenations of ML algorithms and SHAP/PDP tools [5,6,7] by introducing a reusable window-feature-engineering layer and an interpretability protocol with explicit sensitivity rules for correlated exposure features. In this application, VIP, SHAP, and PDP consistently showed that age and adiposity measures are the dominant predictors for individuals. The 60-day PM_2.5_ window is the leading exposure feature, exhibiting a monotonically decreasing marginal profile, while the 7-day window contributes little in the joint models. These results demonstrate the framework’s capacity for hypothesis generation in complex exposure-outcome settings.

The validation results are broadly consistent with prior studies examining PM_2.5_ and blood pressure, while also extending them through systematic multi-window comparison. Howell et al. [35] reported significant interactions between physical activity and PM_2.5_ exposure in relation to hypertension risk, a pattern also observed in the present SHAP interaction analyses. Chen et al. [36] documented positive associations between PM_2.5_ and SBP in rural Chinese populations, consistent with the positive effects identified here for 60-day exposure. Ye et al. [37] demonstrated that both short-term lag effects and seasonal exposure patterns are associated with blood pressure, supporting the rationale for multi-window modeling. The present framework extends these findings by providing a unified analytical structure in which all exposure windows are simultaneously modeled, enabling direct comparison of their relative predictive contributions. In our study, at the univariate level, the mean PM_2.5_ exposure of the hypertensive group was lower than that of the normotensive group in every exposure window. Three possible explanations account for this observation. First, hypertensive participants were, on average, about 10 years older, and both hypertension prevalence and examination dates differed across survey waves and seasons. Second, individuals with known or measured elevated blood pressure tend to reduce strenuous and outdoor activities, and treated patients are more likely to stay indoors. Therefore, their background-monitoring exposure proxy may not accurately reflect personal exposure. Third, the absence of individual time–activity data can attenuate associations. The consistent identification of age, BMI, and waist circumference as the most important predictors aligns with well-established epidemiological evidence [23,24,25,26,27]. The corrected discrimination (AUC 0.69–0.77) is consistent with demographic/clinical risk models of hypertension. Our results support the finding that the 60-day PM_2.5_ × BMI interaction was significant after FDR control.

Several considerations regarding generalizability should be noted. The current validation, which utilized CHNS data with city-level PM_2.5_ exposure estimates, may not capture fine-scale individual exposure variations. However, the modular feature engineering layer is designed to accommodate higher-resolution exposure data. Meanwhile, the negative associations observed for longer exposure windows should be interpreted as associative patterns, as they may reflect unmeasured confounding rather than true protective effects. Finally, the framework was validated on a single Chinese cohort, and the moderate sample size (*n* = 2523 visits) limits the power to detect small exposure effects. External validation in other populations and cohorts is therefore needed to establish broader transportability. Future iterations should also incorporate PM_2.5_ component-specific data and biological pathway markers to strengthen mechanistic inference within the interpretability module.

## 5. Conclusions

This study presents the design, implementation, and initial validation of an interpretable machine learning framework for hypertension risk assessment. This framework systematically integrates multi-window environmental exposure features. When applied to 2523 participant-visits from Beijing CHNS participants, the framework demonstrated realistic predictive performance and yielded the following conclusions:

(1) Multi-window PM_2.5_ features provided a small but statistically significant predictive gain beyond traditional risk factors in the ensemble models.
(2) Window-specific analyses revealed inverse associations that were stronger for longer windows.
(3) The consistent ranking of age, BMI, and waist circumference as top predictors across all five algorithms validates the framework’s alignment and the importance of 60-day PM_2.5_ exposures.

The framework’s modular architecture comprises independent feature engineering, prediction, and interpretability layers, facilitating adaptation to other environmental exposures, health outcomes, and population cohorts. From a public health perspective, its ability to simultaneously quantify risk factor importance and visualize exposure-response patterns supports both population-level risk screening and individualized intervention planning. With respect to multiple comparisons, formal interaction tests were corrected with the Benjamini-Hochberg FDR. However, descriptive comparisons, window-specific estimates, and model-comparison tests are reported with 95% confidence intervals as exploratory analyses without further multiplicity adjustment; isolated borderline *p* values should therefore be interpreted with caution. Future work should prioritize external validation in diverse populations, integration of component-specific PM_2.5_ data, and prospective evaluation of the framework as a clinical decision-support tool.

## Figures and Tables

**Table 1:** Comparison of representative analytical frameworks.

Framework	Exposure Time-Window Handling	Interaction Effects	Operationalizability of Outputs
Ban et al., 2022 [5]	single matrix of pollutant concentrations; no exposure windows	not evaluated	prediction–oriented; limited exposure–response outputs
Li et al., 2023 [6]	single-event/aggregate pollutant levels	SHAP-dependence inspection only	attribution of pollution events to driving factors
Phung et al., 2022 [7]	exposure covariates at one time scale	not evaluated	variable–importance ranking
This framework	five nested cumulative windows produced by a feature-engineering layer; single-window sensitivity models	term interaction tests with FDR control, SHAP/PDP inspection and sex-stratified models	AUC with CI, calibration and Brier score; per–window OR per 10 ug/m^3^; ablation; consensus variable ranking

**Table 2:** Outcome, predictors, validation scheme and evaluation metrics of each analysis.

Analysis	Outcome	Predictors	Validation/Split	Evaluation Metrics
Five ML models	Binary hypertension	Demographic, anthropometric and behavioural variables, the five PM_2.5_ windows	Person-level 80/20 split; 5-fold CV for tuning; SMOTE for training data only; year-based split	AUC, accuracy, sensitivity, specificity, precision, F1, Brier
Window-specific exposure-response models	Binary hypertension	One PM_2.5_ window at a time (per 10 ug/m^3^) + age, sex, BMI, total *MET*	All 2523 participant-visits; complete-case	OR (95% CI) and P
Interaction tests	Binary hypertension	PM_2.5_ × total *MET*/sleep duration/BMI/gender	All participant-visits; Wald tests with Benjamini-Hochberg FDR	Interaction OR (95% CI), P, FDR q
Ablation analysis	Binary hypertension	All predictors with vs. without the five PM_2.5_ windows	Person-level 80/20 split; 5-fold CV for tuning; SMOTE for training data	dAUC with paired bootstrap test
Interpretability (three layers)	Model outputs	All predictors	Test set (*n* = 502)	Global importance, SHAP and PDP

**Table 3:** Characteristics of participant-visits by hypertension status.

	Hytertension (No) *N* = 1884	Hytertension (Yes) *N* = 639	*p*
PM_2.5_, µg/m^3^			
0 day	100.0 ± 87.4	87.2 ± 80.1	<0.001
7 day	100.0 ± 52.8	89.2 ± 43.9	<0.001
15 day	91.8 ± 35.9	86.6 ± 32.9	<0.001
30 day	86.8 ± 31.1	79.5 ± 27.7	<0.001
60 day	79.1 ± 28.4	69.5 ± 24.4	<0.001
met_class:			
met_sit	6.7 ± 5.4	5.8 ± 5.3	<0.001
met_sport	2.3 ± 5.2	2.1 ± 4.3	0.319
met_trans	2.0 ± 9.9	1.1 ± 6.5	0.013
met_work	6.1 ± 10.3	3.8 ± 9.5	<0.001
BMI_class, n (%):			<0.001
Low weight	62 (3.3)	6 (0.9)	
Normal weight	1091 (57.9)	235 (36.8)	
Obesity	110 (5.8)	91 (14.2)	
Over weight	621 (33.0)	307 (48.0)	
Male, n (%)	836 (44.4)	342 (53.5)	<0.001
Sleep duration, h	7.6 ± 1.2	7.6 ± 1.2	0.657
Age, years	48.0 ± 15.1	57.7 ± 12.5	<0.001
Height, cm	164.2 ± 8.4	163.7 ± 8.3	0.203
Weight, kg	65.6 ± 12.0	70.8 ± 11.9	<0.001
BMI, kg/m^2^	24.3 ± 3.8	26.3 ± 3.5	<0.001
Arm circumference, cm	29.0 ± 5.2	29.8 ± 4.2	<0.001
Waist circumference, cm	84.1 ± 11.5	89.8 ± 12.1	<0.001
Hip circumference, cm	95.8 ± 9.9	98.8 ± 10.0	<0.001
Skinfold thickness, mm	20.0 ± 9.1	21.2 ± 8.8	0.006
Total *MET*	24.4 ± 17.1	20.0 ± 14.6	<0.001
SBP, mmHg	119.5 ± 10.6	146.6 ± 12.9	<0.001
DBP, mmHg	76.2 ± 6.8	91.1 ± 9.4	<0.001

Note: *p* values include Welch *t*-test (continuous) or chi-square test (categorical); *N* = 2523; values are mean ± SD or n (%).

**Table 4:** Test performance of the five hypertension classifiers.

Model	AUC (95% CI)	Accuracy	Sensitivity	Specificity	Precision	F1	Brier	C-Intercept	C-Slope
GLM	0.759 (0.715–0.805)	0.675	0.801	0.626	0.456	0.581	0.202	−0.921	0.809
Lasso	0.759 (0.715–0.805)	0.677	0.787	0.634	0.457	0.578	0.203	−0.925	0.822
Decision Tree	0.692 (0.642–0.740)	0.653	0.660	0.651	0.425	0.517	0.233	−0.698	0.125
Random Forest	0.763 (0.718–0.808)	0.685	0.738	0.665	0.462	0.568	0.174	−0.486	0.917
XGBoost	0.768 (0.723–0.813)	0.699	0.752	0.679	0.478	0.584	0.181	−0.310	0.395

Note: C-intercept for Calibration intercept; C-slope for Calibration slope.

**Table 5:** Window-specific associations of PM_2.5_ (per 10 µg/m^3^) with hypertension adjusted for age, sex, BMI and total *MET* using logistical model.

Window	Hypertension OR (95% CI)	*p*
0-day	0.987 (0.976–0.999)	0.036
7-day	0.973 (0.953–0.993)	0.009
15-day	0.982 (0.954–1.010)	0.208
30-day	0.939 (0.908–0.971)	0.0002
60-day	0.897 (0.865–0.931)	<0.0001

## Data Availability

The data and analytic code are available from the corresponding author upon reasonable request.

## Supplementary Materials

The following supporting information can be downloaded at: https://www.mdpi.com/article/10.3390/toxics14100871/s1, Table S1. Results of ablation analysis (models with all five windows vs. no windows); Table S2. Pearson correlations among the five PM_2.5_ exposure windows and variance inflation factors (VIF) in the joint multi-window model.
