# Supplementary material for: An Interpretable Machine Learning Framework Integrating Multi-Window Environmental Exposure for Hypertension Risk Assessment
Source: Toxics. 2026 Sep 30;14(10):871. doi: 10.3390/toxics14100871 (PMC13709448; doi:10.3390/toxics14100871)
Supplement: Supplementary file 1 [file toxics-14-00871-s001.zip › toxics-4504246-supplementary.pdf]

Supplementary Materials

for

An Interpretable Machine Learning Framework Integrating Multi-Window Environmental Exposure for Hypertension Risk Assessment

Ying ZHAO[1*], Kexin Yuan[2]

[1]Department of Chemistry and Chemical Engineering, Jinzhong University, Jinzhong 030619, China; E-Mail: shadowying210@163.com

[2]School of Physical Education, Shanxi University, Taiyuan, Shanxi 030006, China;

*Correspondence: shadowying210@163.com

1. The ablation analysis

The ablation analysis was repeated for all five algorithms(Table S1). All ablation models used the same person-level 80/20 split (2,021 training visits from 1,723 participants; 502 test visits from 431 participants), the same SMOTE protocol applied to the training data only (minority class 498 → 1,523 observations), and the same 5-fold cross-validated hyperparameter search as the main models.

Table S1 Results of ablation analysis (models with all five windows vs. no windows)

Model	With PM2.5 windows	Without PM2.5 windows	ΔAUC	P (paired bootstrap)
GLM	0.759	0.741	+0.018	0.138
Lasso	0.759	0.741	+0.018	0.124
Decision Tree	0.692	0.691	+0.001	0.946
Random Forest	0.763	0.730	+0.033	0.014
XGBoost	0.768	0.731	+0.037	0.010

2. Correlation and variance inflation factors analysis of the nested PM2.5 exposure windows

Table S2. Pearson correlations among the five PM2.5 exposure windows and variance inflation factors (VIF) in the joint multi-window model

Window	0-day	7-day	15-day	30-day	60-day	VIF
0-day	1.000	0.460	0.446	0.384	0.354	1.3
7-day	0.460	1.000	0.838	0.743	0.642	3.5
15-day	0.446	0.838	1.000	0.898	0.797	8.0
30-day	0.384	0.743	0.898	1.000	0.921	13.0
60-day	0.354	0.642	0.797	0.921	1.000	7.0
